# Gender differences in age-related decline in glomerular filtration rates in healthy people and chronic kidney disease patients

**DOI:** 10.1186/1471-2369-11-20

**Published:** 2010-08-23

**Authors:** Rong Xu, Lu-Xia Zhang, Pu-Hong Zhang, Fang Wang, Li Zuo, Hai-Yan Wang

**Affiliations:** 1 Institute of Nephrology, Peking University First Hospital, Beijing, China; 2 Beijing Centers for Diseases Control and Prevention (CDC) & Centers for Preventive Medical Research, Beijing, China

**Keywords:** chronic kidney disease, glomerular filtration rate, gender, aging

## Abstract

**Background:**

Since men with chronic kidney disease (CKD) progress faster than women, an accurate assessment of CKD progression rates should be based on gender differences in age-related decline of glomerular filtration rate (GFR) in healthy individuals.

**Methods:**

A Chinese sample population from a stratified, multistage, and clustered CKD screening study was classified into healthy, at-risk, and CKD groups. The gender differences in estimated GFR (eGFR) and age-related eGFR decline were calculated for each group after controlling for blood pressure, fasting glucose levels, serum lipids levels, education level, and smoking status. After referencing to the healthy group, gender-specific multivariate-adjusted rates of decline in eGFR and differences in the rates of decline were calculated for both CKD and at-risk groups.

**Results:**

The healthy, at-risk, and CKD groups consisted of 4569, 7434, and 1573 people, respectively. In all the 3 groups, the multivariate-adjusted eGFRs in men were lower than the corresponding eGFRs in women. In addition, in the healthy and at-risk groups, the rates of decline in eGFR in men were lower than the corresponding rates of decline in women (healthy group: 0.51 mL·min^-1^·1.73 m^-2^·yr^-1 ^*vs*. 0.74 mL·min^-1^·1.73 m^-2^·yr^-1 ^and at-risk group: 0.60 mL·min^-1^·1.73 m^-2^·yr^-1 ^*vs*. 0.73 mL·min^-1^·1.73 m^-2^·yr^-1^). However, in the CKD group, the rates of decline in eGFR in men were similar to those in women (0.96 mL·min^-1^·1.73 m^-2^·yr^-1 ^*vs*. 0.91 mL·min^-1^·1.73 m^-2^·yr^-1^). However, after referencing to the healthy group, the rates of decline in eGFR in men in the at-risk and CKD groups were greater faster than the corresponding rates in women (at-risk group: 0.10 mL·min^-1^·1.73 m^-2^·yr^-1 ^*vs*. -0.03 mL·min^-1^·1.73 m^-2^·yr^-1 ^and CKD group: 0.44 mL·min^-1^·1.73 m^-2^·yr^-1 ^*vs*. 0.15 mL·min^-1^·1.73 m^-2^·yr^-1^).

**Conclusion:**

To accurately assess gender differences in CKD progression rates, gender differences in age-related decline in GFR should be considered.

## Background

Men with chronic kidney disease (CKD) progress to end-stage CKD at a faster rate than women[1,2]. This gender-specific difference cannot be fully explained on the basis of differences in blood pressure, glucose metabolism, or serum cholesterol levels. It is speculated that this difference may be related to gender-specific differences in glomerular structure, hemodynamic condition, activity of local cytokines and hormones, gene expression, and/or the effects of sex hormones on kidney cells[3,4]. However, these differences also exist in healthy genders[5,6] and cause gender difference in age-related loss of glomerular filtration rate (GFR) in healthy population[7-9]. Thus, an accurate assessment of the gender-specific CKD progression rate should be based on the gender differences in age-related decline in GFR in healthy individuals. In addition, some previous studies did not observe a faster progression of CKD in men[10,11]. The gender-specific difference in CKD progression rates reflect not only biological differences but also differences in environmental, socioeconomic, lifestyle, and health care factors, which are not usually considered as risk factors for CKD and its progression[12,13]. Therefore, to determine the effect of gender alone on CKD progression, these factors should be adjusted. Therefore, we used a randomly sampled population from a well organized CKD screening study to determine gender-specific difference in CKD progression rates after adjusting for the normal age-related GFR decline and after controlling for potential confounding variables, such as health status, socioeconomic status, and lifestyle behaviors.

## Methods

### Participants and methods

We extracted a database of subjects from a stratified CKD cluster sampling study performed in Beijing in 2006[14]. A total of 15,370 adults were selected from 53 primary sampling units and invited to participate. Women needed to be oversampled to obtain sufficient data due to the high male to female sex ratio in China. After weighting, the ratio of men to women in the study was 1:1.18. Ultimately, 13,925 subjects completed the survey and physical examination. Overall, the enrollment rate was 90.6% (92.1% of females and 89.6% of males). The ethics committees in Peking university first hospital approved this study. After obtaining informed consent from all subjects, they completed a screening questionnaire to collect information about socioeconomic status (*e.g*., gender, age, and education), personal and family health history, and lifestyle behaviors (*e.g*., smoking). Laboratory measurements also were made by well-trained professionals using uniform standards.

Serum creatinine (S_cr_) was measured by using a kinetic-rate method described by Jaffe. The glomerular filtration rate was estimated by using a modified Modification of Diet in Renal Disease (MDRD) Study formula[15]: estimated GFR (eGFR, mL·min^-1^·1.73 m^-2^) = 175 × standard (S_cr_)^-1.234 ^× age^-0.179 ^× 0.79 (if female).

Urine albumin and creatinine measurements were made from morning spot urine samples. Urine albumin was measured by using an immunoturbidimetric assay (Audit Diagnostics, Cork, Ireland) and urine creatinine was measured by using Jaffe's kinetic method on a Hitachi 7170 chemistry analyzer (Tokyo, Japan). Albuminuria was diagnosed if the urine albumin/creatinine ratio was ≥17 mg·g^-1 ^for men or ≥25 mg·g^-1 ^for women[16] and proteinuria was diagnosed if this ratio was ≥300 mg·g^-1 ^for either men or women.

Hypertension was defined as measurement of systolic blood pressure ≥140 mmHg or diastolic blood pressure ≥ 90 mmHg, having been diagnosed with hypertension at least twice, or taking an antihypertensive medication at the time of the survey.

Diabetes was defined as measurement of a fasting glucose level > 6.9 mmol·L^-1^, a previous diagnosis of diabetes, or taking insulin or anti-diabetic medication at the time of the survey.

Dyslipidemia was defined as measurement of total cholesterol (TC) ≥6.22 mmol·L^-1^, triglyceride (TG) ≥2.26 mmol·L^-1^, high-density lipoprotein (HDL) < 1.04 mmol·L^-1^, or low-density lipoprotein (LDL) ≥4.14 mmol·L^-1^; a previous diagnosis of dyslipidemia; or taking lipid-lowering medication at the time of the survey.

Obesity was defined as having a body mass index (BMI) ≥28 kg·m^-2 ^(BMI = weight/(height^2^)).

Individuals whose eGFR was > 200 mL·min^-1^·1.73 m^-2 ^(n = 33) or < 15 mL·min^-1^·1.73 m^-2 ^(n = 57) were excluded from this study. Participants were divided into 3 groups according to their health status: those with albuminuria or eGFR < 60 mL·min^-1^·1.73 m^-2 ^were placed in the CKD group; those without CKD but with hypertension, diabetes, dyslipidemia, or obesity were placed in the at-risk group; those without CKD or other medical conditions were placed in the healthy group. We also used proteinuria as a definition of CKD and performed a separate statistical analysis as described below because currently there is no consensus about defining CKD by microalbuminuria or macroalbuminuria. Some authors only consider microalbuminuria to be a risk factor for CKD such as (pre)hypertension or diabetes and its reproducibility of microalbuminuria tests is limited [34].

### Statistical analyses

#### General characteristics in each health status group

Sociodemographic characteristics (age, highest education level), health indicators (BMI, fasting glucose level, serum lipid levels, eGFR), and lifestyle behaviors (smoking) of each gender were described by descriptive statistics. The differences in these variables among genders were examined by using chi-square statistics for categorical variables and Student's t-test for continuous values.

#### Gender-specific adjusted eGFRs in each health status group

For each health status group, a linear model was constructed with eGFR as the dependent variable, gender as the independent variable, and confounding factors (systolic blood pressure, diastolic blood pressure, BMI, fasting glucose level, serum lipid levels, highest education level, and smoking) as covariates. This model was used to calculate the least squares means of eGFR (adjusted eGFR) and to compare eGFRs between genders within each health status group.

#### Gender-specific rates of decline of eGFR in each health status group

A linear model was constructed for each health status group. In these models, the dependent variable was eGFR and the independent variables were age, gender, and interaction of age and gender. These models were used to calculate the gender-specific rates of decline of eGFR and to test gender-specific differences. Subsequently, confounding factors were added to these models as covariates to calculate the adjusted gender-specific rates of decline of eGFR.

#### Adjusted gender-specific rates of decline of eGFR (referenced to healthy group) in CKD and at-risk groups

A linear regression model was constructed for each gender. In these models, the variables were (1) eGFR (dependent variable); (2) age, health status, and interaction of age and health status (independent variables); and (3) the aforementioned confounding factors (covariates). This model was used to calculate the adjusted gender-specific rates of decline of eGFR (referenced to the healthy group) for the CKD and at-risk groups. In addition, a linear regression model was constructed with the following variables: (1) eGFR (dependent variable); (2) age, health status, gender, and interactions of age and health status, age and gender, health status and gender, age and health status and gender (independent variables); and (3) the aforementioned confounding factors (covariates). This model was used to test for statistically significant differences between men and women for the adjusted gender-specific rates of decline of eGFR (referenced to the healthy group) for the CKD and at-risk groups.

All statistical analyses were performed using Statistical Analysis System software, version 9.0 (SAS Institute Inc., Cary, NC, USA). Tests for association were considered to be statistically significant when the p-values were less than 0.05.

## Results

The healthy, at-risk, and CKD groups consisted of 4569, 7434, and 1573 people, respectively. There were 1396 individuals (10.04%) with microalbuminuria, 104 (0.75%) with proteinuria, and 261 (1.88%) with eGFR < 60 mL·min^-1^·1.73 m^-2^. In the healthy group, men had higher BMIs, blood pressures, fasting glucose levels, serum lipid levels, and lower education levels than women of similar ages. In the at-risk group, men were younger, had higher diastolic blood pressures, TG levels, and education levels but lower levels of LDL, TC, and HDL than women. In the CKD group, men had higher blood pressures, TG levels, and education levels but lower HDL levels than women. In each health status group, men were more likely to be smokers. Compared to individuals in the healthy group, those in the at-risk and CKD groups were older and fatter and had higher blood pressures and serum lipid levels. For men, the prevalence of smoking was similar in all health status groups and the CKD group had significantly lower education levels than the healthy or at-risk groups. For women, the at-risk and CKD groups had significantly lower education levels and more smokers than the healthy group (Table 1).

**Table 1:** Characteristics of study participants in healthy, at-risk, and CKD groups

	Healthy group		At-risk group		CKD group	
	male(n = 1311)	female(n = 3258)	male(n = 3338)	female(n = 4096)	male(n = 676)	female(n = 897)
**Age (years)**	38.58 ± 13.23	38.00 ± 10.59	47.25 ± 13.14*#	50.54 ± 12.07&	51.62 ± 14.64#	50.92 ± 14.56&
**BMI (kg·m^-2^)**	22.93 ± 2.54*	22.44 ± 2.56	26.05 ± 3.32#	26.12 ± 3.72&	26.30 ± 3.81#	25.98 ± 4.11&
**FG (mmol·L^-1^)**	5.04 ± 0.45*	4.95 ± 0.43	5.72 ± 1.69#	5.79 ± 1.72&	6.28 ± 2.39#	6.09 ± 2.38&
**HDL (mmol·L^-1^)**	1.36 ± 0.25*	1.48 ± 0.27	1.19 ± 0.30*#	1.34 ± 0.33&	1.24 ± 0.33*#	1.34 ± 0.31&
**LDL (mmol·L^-1^)**	2.79 ± 0.69*	2.66 ± 0.64	3.29 ± 0.93*#	3.40 ± 0.94&	3.27 ± 1.03#	3.25 ± 0.97&
**TC (mmol·L^-1^)**	4.41 ± 0.71*	4.37 ± 0.68	4.97 ± 0.99*#	5.11 ± 1.01&	5.02 ± 1.04#	5.05 ± 1.06&
**TG (mmol·L^-1^)**	0.97 ± 0.44*	0.82 ± 0.37	1.93 ± 1.59*#	1.60 ± 1.20&	1.99 ± 1.78*#	1.65 ± 1.37&
**DBP (mmHg)**	76.07 ± 7.37*	72.83 ± 7.58	86.16 ± 10.98*#	84.27 ± 11.47&	88.75 ± 13.45*#	84.52 ± 12.93&
**SBP (mmHg)**	121.45 ± 9.24*	113.78 ± 10.97	137.23 ± 18.45#	136.92 ± 22.11&	144.51 ± 22.85*#	140.10 ± 26.68&
**Low education (%)**	34.23*	24.09	34.18*	46.67&	42.94*#	48.59&
**Smoker (%)**	56.29*	3.13	59.04*	5.74&	54.59*	7.15&
**eGFR** **(mL·min^-1^·1.73 m^-2^)**	102.07 ± 19.23*	107.72 ± 20.60	94.74 ± 19.54*#	98.24 ± 19.89&	88.49 ± 25.98*#	92.32 ± 28.72&
**adj-eGFR** **(mL·min^-1^·1.73 m^-2^)**	95.64 ± 0.60 *	102.08 ± 0.41	96.51 ± 0.34*	101.97 ± 0.34	92.28 ± 0.74*#	95.85 ± 0.67&

**BMI**: body mass index; **FG**: fasting glucose; **HDL**: high-density lipoprotein; **LDL**: low-density lipoprotein; **TC: **total cholesterol; **TG**: triglyceride; **DBP: **diastolic blood pressure; **SBP**: systolic blood pressure; **eGFR**: estimated glomerular filtration rate; **adj-eGFR**: eGFR adjusted for age, blood pressure, fasting glucose level, serum lipid level, education level, and smoking

*: Compared to women in the same health status group, p < 0.05

#: Compared to men in the healthy group, p < 0.05

&: Compared to women in the healthy group, p < 0.05

The gender differences of adjusted eGFRs in each health status and age groups are shown in Table 2. In the healthy group, men younger than 60 years old had significantly lower eGFRs than women of similar ages. After age 60, this relationship reversed but the difference was not significant. In the at-risk group, men younger than 70 years old had significantly lower eGFRs than women of similar ages. However, in the CKD group, both men and women had similar eGFRs in each age group.

**Table 2:** Gender differences of adjusted eGFR^† ^(mL·min^-1^·1.73 m^-2^, mean ± standard error) in each age group in healthy, at-risk, and CKD groups

Age group		Healthy group		At-risk group		CKD group	
		
		Men	Women	Men	Women	Men	Women
**18-30 years**	**Creatinine**	83.94 ± 0.59*	64.41 ± 0.32	84.70 ± 0.66*	64.55 ± 0.70	86.72 ± 7.17*	66.61 ± 1.69
	**eGFR**	109.13 ± 1.03*	118.89 ± 0.76	107.63 ± 1.27*	117.87 ± 1.63	111.38 ± 4.70	116.48 ± 3.30
	**adj-eGFR**	107.83 ± 1.07*	119.56 ± 0.75	107.59 ± 1.22*	117.94 ± 1.81	108.39 ± 4.13	118.52 ± 3.34
	**N**	**384**	**729**	**328**	**162**	**47**	**69**
							
**30-40 years**	**Creatinine**	81.79 ± 0.63*	65.47 ± 0.28	84.76 ± 0.51*	66.29 ± 0.41	87.96 ± 1.57*	68.45 ± 1.41
	**eGFR**	105.13 ± 1.04*	109.00 ± 0.57	100.89 ± 0.97*	107.14 ± 0.86	104.27 ± 2.41	106.62 ± 2.35
	**adj-eGFR**	104.50 ± 1.08*	109.22 ± 0.58	101.42 ± 0.93*	106.62 ± 0.97	106.25 ± 2.73	105.11 ± 2.27
	**N**	**344**	**1077**	**556**	**514**	**90**	**126**
							
**40-50 years**	**Creatinine**	82.84 ± 0.61*	65.89 ± 0.30	84.82 ± 0.35*	66.45 ± 0.27	88.83 ± 1.97*	72.87 ± 1.50
	**eGFR**	98.48 ± 0.94*	103.93 ± 0.61	95.82 ± 0.54*	102.65 ± 0.54	93.69 ± 1.64	96.38 ± 1.57
	**adj-eGFR**	98.66 ± 1.09*	103.88 ± 0.58	96.13 ± 0.58*	102.42 ± 0.52	94.85 ± 1.83	95.56 ± 1.52
	**N**	**310**	**1026**	**1053**	**1308**	**175**	**248**
							
**50-60 years**	**Creatinine**	84.13 ± 0.79*	68.20 ± 0.51	86.19 ± 0.39*	68.85 ± 0.28	92.02 ± 1.91*	76.72 ± 2.06
	**eGFR**	93.47 ± 1.15*	96.31 ± 0.95	90.91 ± 0.57*	94.94 ± 0.49	87.49 ± 1.74	90.32 ± 1.86
	**adj-eGFR**	93.26 ± 1.29*	96.42 ± 0.92	90.89 ± 0.60*	94.95 ± 0.49	88.21 ± 1.92	89.71 ± 0.75
	**N**	**184**	**340**	**845**	**1212**	**170**	**204**
							
**60-70 years**	**Creatinine**	83.57 ± 1.37*	70.74 ± 1.28	87.53 ± 0.61*	70.14 ± 0.41	101.27 ± 3.22*	85.45 ± 4.76
	**eGFR**	91.14 ± 1.87	88.76 ± 2.08	86.13 ± 0.78*	89.83 ± 0.70	76.30 ± 2.26	80.74 ± 2.45
	**adj-eGFR**	91.20 ± 2.02	88.70 ± 2.08	86.03 ± 0.88*	89.76 ± 0.67	75.54 ± 2.43	81.63 ± 2.19
	**N**	**62**	**59**	**351**	**580**	**100**	**125**
							
**70-years**	**Creatinine**	84.92 ± 2.42*	72.16 ± 1.77	88.77 ± 0.79*	72.72 ± 0.55	109.46 ± 2.99*	86.89 ± 1.95
	**eGFR**	87.46 ± 3.11	84.33 ± 2.66	82.48 ± 0.95	83.75 ± 0.85	67.07 ± 1.94	71.39 ± 2.14
	**adj-eGFR**	90.17 ± 3.10	81.62 ± 3.10	82.69 ± 1.06	83.50 ± 0.84	65.82 ± 2.30	71.83 ± 1.97
	**N**	**27**	**27**	**205**	**320**	**94**	**125**

^†^: Controlled for age, blood pressure, fasting glucose level, serum lipid level, education level, and smoking frequency

*: Compared to women in the same health status group, p < 0.05

As shown in Table 3, the unadjusted rates of decline of eGFR were 0.51 mL·min^-1^·1.73 m^-2^·yr^-1 ^for men and 0.75 mL·min^-1^·1.73 m^-2^·yr^-1 ^for women in the healthy group (p < 0.05), 0.53 mL·min^-1^·1.73 m^-2^·yr^-1 ^for men and 0.67 mL·min^-1^·1.73 m^-2^·yr^-1 ^for women in the at-risk group (p < 0.05), and 0.89 mL·min^-1^·1.73 m^-2^·yr^-1 ^for men and 0.88 mL·min^-1^·1.73 m^-2^·yr^-1 ^for women in the CKD group (p > 0.05). After adjusting for covariates, the rates of decline of eGFR were 0.51 mL·min^-1^·1.73 m^-2^·yr^-1 ^for men and 0.74 mL·min^-1^·1.73 m^-2^·yr^-1 ^for women in the healthy group (p < 0.05), 0.60 mL·min^-1^·1.73 m^-2^·yr^-1 ^for men and 0.73 mL·min^-1^·1.73 m^-2^·yr^-1 ^for women in the at-risk group (p < 0.05), and 0.96 mL·min^-1^·1.73 m^-2^·yr^-1 ^for men and 0.91 mL·min^-1^·1.73 m^-2^·yr^-1 ^for women in the CKD group (p > 0.05). In addition, we calculated the variation in the rates of decline of eGFR before and after the age of 50. However, there was no significant difference for either gender in each health status group.

**Table 3:** Differences in rates of decline in eGFR (mL·min^-1^·1.73 m^-^^2^·yr^-1^, mean ± standard error) between genders and between groups

	Health group (n = 4569)		Risk group (n = 7434)		CKD group (n = 1573)	
	
	Male (n = 1311)	Female (n = 3258)	Male (n = 3338)	Female (n = 4096)	Male (n = 676)	Female (n = 897)
**Unadjusted rates of decline in eGFR**	0.51 ± 0.04*	0.75 ± 0.03	0.53 ± 0.02*	0.67 ± 0.02	0.89 ± 0.06	0.88 ± 0.06
**Covariate-adjusted rates of decline in eGFR^†^**	0.52 ± 0.04*	0.75 ± 0.04	0.62 ± 0.03*	0.71 ± 0.03	0.94 ± 0.06	0.90 ± 0.08
**Covariate-adjusted rates of decline in eGFR^† ^(referenced to healthy group)**	-	-	0.10 ± 0.05&	-0.03 ± 0.04	0.44 ± 0.06&	0.15 ± 0.06&
**Gender difference in covariate-adjusted rates of decline in eGFR^† ^(referenced to healthy group)**	-		0.10 ± 0.06		0.29 ± 0.09#	

*: Compared to women in the same health status group, p < 0.05

&: Compared to healthy group in the same gender, p < 0.05

#: p < 0.05

^†^: Controlled for age, blood pressure, fasting glucose level, serum lipid level, education level, and smoking frequency

Table 3 also shows that the adjusted rates of decline of eGFR (referenced to the healthy group) was 0.10 mL·min^-1^·1.73 m^-2^·yr^-1 ^(p < 0.05) for men and -0.03 mL·min^-1^·1.73 m^-2^·yr^-1 ^(p > 0.05) for women in the at-risk group and 0.44 mL·min^-1^·1.73 m^-2^·yr^-1 ^(p < 0.05) for men and 0.15 mL·min^-1^·1.73 m^-2^·yr^-1 ^(p < 0.05) for women in the CKD group. The gender difference of the adjusted rate of decline of eGFR (referenced to the healthy group) was statistically significant for the CKD group (0.29 mL·min^-1^·1.73 m^-2^·yr^-1^, p < 0.05) but not the at-risk group (0.10 mL·min^-1^·1.73 m^-2^·yr^-1^, p > 0.05).

When we used proteinuria to define CKD, we obtained different results for the CKD group (Table 4). For men, the difference of the adjusted rate of decline of eGFR between the CKD and healthy groups increased from 0.44 mL·min^-1^·1.73 m^-2^·yr^-1 ^(p < 0.05) (Table 3) to 0.51 mL·min^-1^·1.73 m^-2^·yr^-1 ^(p < 0.05) (Table 4). For women, this difference increased from 0.15 mL·min^-1^·1.73 m^-2^·yr^-1 ^(p < 0.05) (Table 3) to 0.20 mL·min^-1^·1.73 m^-2^·yr^-1 ^(p < 0.05) (Table 4). However, the gender difference of the adjusted rate of decline of eGFR in the CKD group did not show any significant change (0.29 mL·min^-1^·1.73 m^-2^·yr^-1 ^*vs*. 0.31 mL·min^-1^·1.73 m^-2^·yr^-1^). Moreover, these changes were quantitative but not qualitative, which was partly due to the significantly smaller sample size in the CKD group when proteinuria was used to define CKD.

**Table 4:** Differences in rates of decline in eGFR (mL·min^-1^·1.73 m^-2^·yr^-1^, mean ± standard error) within genders and between groups when proteinuria was used to define CKD

	Health group (n = 4660)		Risk group (n = 8566)		CKD group (n = 341)	
	
	Male (n = 1353)	Female (n = 3307)	Male (n = 3836)	Female (n = 4730)	Male (n = 130)	Female (n = 211)
**Unadjusted rates of decline in eGFR**	0.52 ± 0.04*	0.75 ± 0.03	0.52 ± 0.02*	0.67 ± 0.02	0.97 ± 0.08	0.91 ± 0.07
**Covariate-adjusted rates of decline in eGFR^†^**	0.52 ± 0.04*	0.74 ± 0.04	0.61 ± 0.03*	0.71 ± 0.03	1.03 ± 0.08	0.97 ± 0.10
**Covariate-adjusted rates of decline in eGFR^† ^(referenced to healthy group)**	-	-	0.10 ± 0.04&	-0.03 ± 0.04	0.51 ± 0.08&	0.20 ± 0.07&
**Gender difference of covariate adjusted rates of decline in eGFR^† ^(referenced to healthy group)**	-		0.09 ± 0.06		0.31 ± 0.10#	

## Discussion

Currently information about gender differences in GFRs in healthy individuals is limited and conflicting. For example, our observation that men younger than 60 years old had lower eGFRs than women of similar ages is both consistent[17] and inconsistent[7,18-21] with previous studies. In addition, the information about gender differences in age-related rates of decline of GFR is not very consistent. For instance, our observation that healthy men had lower rates of decline of eGFR than healthy women is in agreement with some previous studies[8,22] but not others[7,21,23]. Unlike the participants in previous studies, who were voluntary kidney donors[7,8,18,20,21,23] or local residents[17,19], participants in this study were selected from a prior stratified cluster sampling study, which represented the study population well and minimized selection bias.

Some earlier studies showed an increase in the rates of decline of GFR in healthy individuals older than 40 or 50 years old[18,19,21] or a constant GFR for women younger than 50 years old[23]. However, other studies observed a linear relationship between GFR and age[8,20]. In this study, we did not find any significant difference in the rate of decline of eGFR before or after the age of 50 for either gender. In addition, although we observed that older healthy subjects had significantly lower eGFRs than young healthy subjects, the difference was not as large as we expected. This finding is consistent with the hypothesis that kidney function may not decrease with age but rather with age-related increases in the incidence of degenerative diseases and risk factors for vascular and kidney disease. In most former studies, few study participants were elderly and exclusion criteria were more relaxed than in our study. Any study about the rate of decline of GFR in healthy individuals that does not exclude all possible confounding factors that can affect the GFR, which are common in the elderly, would be expected to show an increased rate of decline of GFR in the elderly.

Our observation that the rate of decline of eGFR in men was slower in the healthy group but faster in the CKD group could be explained by epidemiology studies. In general, men have a higher incidence and prevalence of end-stage renal disease (ESRD) than women [24,25]; however, most population-based CKD screening studies show that men have a similar[14,26] or lower[27-29] prevalence of CKD than women. This discrepancy suggests 2 potential mechanisms for the rate of decline of GFR between non-CKD and CKD stages. One possible explanation is that renal function is preserved as healthy individuals age at the expense of a gradual decrease in the renal functional reserve[30]. Men may have a higher renal functional reserve to compensate for the accumulation of sclerotic glomeruli during aging. Indeed, previous studies show that healthy men have a greater ability to maintain their GFRs by increasing the filtration fraction than women[31]. In addition, gender-specific differences in glomerular hemodynamic may contribute to differences in age-related decline of GFRs. Another possible explanation is that although the whole-body levels[6] and renal levels[32] of nitric oxide, a vasodilator, are higher in healthy premenstrual women than healthy men, which may cause a higher GFR in premenstrual women, the renal vasculature of men may become increasingly dependent on nitric oxide with age compared with that of women[5]. If this is true, then any renal disease that interferes with nitric oxide production may cause kidney damage to progress more quickly in men than in women.

This study had 3 potential limitations. First, the use of estimated GFR to assess renal function in healthy individuals could be prone to measurement bias[17]. However, when we used the Cockcroft-Gault formula, which was developed from healthy individuals and is more accurate than the MDRD formula[17], to assess renal function in healthy individuals, men still had lower eGFRs and slower age-related rates of decline of eGFR than women. Furthermore, when we excluded individuals younger than 30 years old (who are healthier), our results were similar. Second, we had to estimate GFRs rather than measure them directly and only a single measurement of serum creatinine was available for each participant. In addition, morning spot urine samples were collected rather than first morning void urine samples. It is known that first morning void urine samples may provide more accurate results of albuminuria [33] and that the reproducibility of microalbuminuria tests is limited (60-70%) [34]. Despite these limitations, the procedures used in this study were chosen due to the expense and inherent difficulties of directly measuring GFRs and collecting first-morning void urine samples and many blood and urine samples in a study with more than 10,000 participants. Third, estimating age-related rates of decline of eGFR from a cross-sectional study undoubtedly produced bias; however, appropriate mathematical models were used to reduce the bias as much as possible. In addition, since men had a higher risk of cardiovascular disease and death with increasing age, especially those with chronic renal insufficiency, the regression line of GFR against age would deviate from its actual trend for a higher non-response rate in men than in women. However, in this study, men had a similar response rate as that in women (89.6% vs. 92.1%). Moreover, if a correction were made for the higher non-response rate in unhealthy men, who tended to have lower GFRs than participants who responded, then the gender differences in the rate of decline of eGFR would be greater.

## Conclusion

There are gender differences in the decline in GFR and among different health statuses. To accurately evaluate the gender differences in CKD progression rate, the baseline gender differences in age-related decline in GFR in healthy individuals should be considered.

## Competing interests

The authors declare that they have no competing interests.

## Authors' contributions

RX analyzed data and wrote the paper.

LXZ, PHZ, and FW performed research.

LZ and HYW designed research.

All authors read and approved the final manuscript.

## Pre-publication history

The pre-publication history for this paper can be accessed here:

http://www.biomedcentral.com/1471-2369/11/20/prepub

## References

[B1] Neugarten J Acharya A Silbiger SR Effect of gender on the progression of nondiabetic renal disease: a meta-analysis J Am Soc Nephrol 2000 11 319 29 10665939 10.1681/ASN.V112319

[B2] Silbiger SR Neugarten J The impact of gender on the progression of chronic renal disease Am J Kidney Dis 1995 25 515 33 10.1016/0272-6386(95)90119-1 7702046

[B3] Silbiger SR Neugarten J The role of gender in the progression of renal disease Adv Ren Replace Ther 2003 10 3 14 10.1053/jarr.2003.50001 12616458

[B4] Lu H Klaassen C Gender differences in mRNA expression of ATP-binding cassette efflux and bile acid transporters in kidney, liver, and intestine of 5/6 nephrectomized rats Drug Metab Dispos 2008 36 16 23 10.1124/dmd.107.014845 17855625

[B5] Sofia Ahmed B Naomi Fisher DL Norman K Hollenberg. Gender and the Renal Nitric Oxide Synthase System in Healthy Humans Clin J Am Soc Nephrol 2007 916 931 10.2215/CJN.00110107 17702739

[B6] Forte P Kneale BJ Milne E Evidence for a difference in nitric oxide biosynthesis between healthy women and men Hypertension 1998 32 730 4 9774371 10.1161/01.hyp.32.4.730

[B7] Barai S Bandopadhayaya GP Patel CD Do healthy potential kidney donors in india have an average glomerular filtration rate of 81.4 ml/min? Nephron Physiol 2005 101 21 6 10.1159/000086038 15925908

[B8] Rule AD Gussak HM Pond GR Measured and estimated GFR in healthy potential kidney donors Am J Kidney Dis 2004 43 112 9 10.1053/j.ajkd.2003.09.026 14712434

[B9] Fliser D Zeier M Nowack R Renal functional reserve in healthy elderly subjects J Am Soc Nephrol 1993 3 1371 7 8439649 10.1681/ASN.V371371

[B10] D'Amico G Influence of clinical and histological features on actuarial renal survival in adult patients with idiopathic IgA nephropathy, membranous nephropathy, and membranoproliferative glomerulonephritis: survey of the recent literature Am J Kidney Dis 1992 20 315 23 1415198 10.1016/s0272-6386(12)70293-7

[B11] Coggins CH Breyer Lewis J Caggiula AW Differences between women and men with chronic renal disease Nephrol Dial Transplant 1998 13 1430 7 10.1093/ndt/13.6.1430 9641172

[B12] Orth SR Hallan SI Smoking: a risk factor for progression of chronic kidney disease and for cardiovascular morbidity and mortality in renal patients--absence of evidence or evidence of absence? Clin J Am Soc Nephrol 2008 3 226 36 10.2215/CJN.03740907 18003763

[B13] Taal MW Brenner BM Predicting initiation and progression of chronic kidney disease: Developing renal risk scores Kidney Int 2006 70 1694 705 10.1038/sj.ki.5001794 16969387

[B14] Zhang L Zhang P Wang F Prevalence and factors associated with CKD: a population study from Beijing Am J Kidney Dis 2008 51 373 84 10.1053/j.ajkd.2007.11.009 18295053

[B15] Ma YC Zuo L Chen JH Modified glomerular filtration rate estimating equation for Chinese patients with chronic kidney disease J Am Soc Nephrol 2006 17 2937 44 10.1681/ASN.2006040368 16988059

[B16] Mattix HJ Hsu CY Shaykevich S Use of the albumin/creatinine ratio to detect microalbuminuria: implications of sex and race J Am Soc Nephrol 2002 13 1034 9 11912263 10.1681/ASN.V1341034

[B17] Vervoort G Willems HL Wetzels JF Assessment of glomerular filtration rate in healthy subjects and normoalbuminuric diabetic patients: validity of a new (MDRD) prediction equation Nephrol Dial Transplant 2002 17 1909 13 10.1093/ndt/17.11.1909 12401845

[B18] Grewal GS Blake GM Reference data for 51Cr-EDTA measurements of the glomerular filtration rate derived from live kidney donors Nucl Med Commun 2005 26 61 5 10.1097/00006231-200501000-00010 15604949

[B19] Klingensmith WC Briggs DE Smith WI Technetium-99 m-MAG3 renal studies: normal range and reproducibility of physiologic parameters as a function of age and sex J Nucl Med 1994 35 1612 7 7931658

[B20] Slack TK Wilson DM Normal renal function: CIN and CPAH in healthy donors before and after nephrectomy Mayo Clin Proc 1976 51 296 300 1263599

[B21] Granerus G Aurell M Reference values for 51Cr-EDTA clearance as a measure of glomerular filtration rate Scand J Clin Lab Invest 1981 41 611 6 10.3109/00365518109090505 6801756

[B22] Shyh-Dye Lee Rong-Bin Chuang Chia-Ming Li Wen-Han Pan Aging of Renal Function Estimation in Elderly Community Dwellers Experiences in Taiwanese Population Tai J Geront S Med 2006 1 159 172

[B23] Berg UB Differences in decline in GFR with age between males and females. Reference data on clearances of inulin and PAH in potential kidney donors Nephrol Dial Transplant 2006 21 2577 82 10.1093/ndt/gfl227 16720595

[B24] Iseki K Nakai S Shinzato T Increasing gender difference in the incidence of chronic dialysis therapy in Japan Ther Apher Dial 2005 9 407 11 10.1111/j.1744-9987.2005.00318.x 16202016

[B25] USRDS USRDS 2007 Annual Data Report. National Institutes of Health, National Institute of Diabetes and Digestive and Kidney Diseases 2007 84

[B26] Coresh J Astor BC Greene T Prevalence of chronic kidney disease and decreased kidney function in the adult US population: Third National Health and Nutrition Examination Survey Am J Kidney Dis 2003 41 1 12 10.1053/ajkd.2003.50007 12500213

[B27] Chadban SJ Briganti EM Kerr PG Prevalence of kidney damage in Australian adults: The AusDiab kidney study J Am Soc Nephrol 2003 14 S131 8 10.1097/01.ASN.0000070152.11927.4A 12819318

[B28] Nitsch D Felber Dietrich D von Eckardstein A Prevalence of renal impairment and its association with cardiovascular risk factors in a general population: results of the Swiss SAPALDIA study Nephrol Dial Transplant 2006 21 935 44 10.1093/ndt/gfk021 16390852

[B29] Viktorsdottir O Palsson R Andresdottir MB Prevalence of chronic kidney disease based on estimated glomerular filtration rate and proteinuria in Icelandic adults Nephrol Dial Transplant 2005 20 1799 807 10.1093/ndt/gfh914 15928100

[B30] Fuiano G Sund S Mazza G Renal hemodynamic response to maximal vasodilating stimulus in healthy older subjects Kidney Int 2001 59 1052 8 10.1046/j.1523-1755.2001.0590031052.x 11231360

[B31] Miller JA Anacta LA Cattran DC Impact of gender on the renal response to angiotensin II Kidney Int 1999 55 278 85 10.1046/j.1523-1755.1999.00260.x 9893137

[B32] Reckelhoff JF Hennington BS Moore AG Gender differences in the renal nitric oxide (NO) system: dissociation between expression of endothelial NO synthase and renal hemodynamic response to NO synthase inhibition Am J Hypertens 1998 11 97 104 10.1016/S0895-7061(97)00360-9 9504456

[B33] Witte EC Lambers HHJ de Zeeuw D First morning voids are more reliable than spot urine samples to assess microalbuminuria J Am Soc Nephrol 2009 20 436 43 10.1681/ASN.2008030292 19092125 PMC2637059

[B34] Coresh J Astor BC Greene T Prevalence of chronic kidney disease and decreased kidney function in the adult US population: Third National Health and Nutrition Examination Survey Am J Kidney Dis 2003 41 1 12 10.1053/ajkd.2003.50007 12500213

